# Photochemically Active Fluorophore–DNA/RNA Conjugates for Cellular Imaging of Nucleic Acids by Readout in Electron Microscopy

**DOI:** 10.1002/open.201300017

**Published:** 2013-06-21

**Authors:** Carolin Holzhauser, Sabrina Kracher, Moritz M Rubner, Wolfgang Schmucker, Hans-Achim Wagenknecht, Ralph Witzgall

**Affiliations:** [a] Institute of Organic Chemistry, Karlsruhe Institute of Technology (KIT)Fritz-Haber-Weg 6, 76131 Karlsruhe (Germany) E-mail: Wagenknecht@kit.edu; [b] Institute of Molecular and Cellular Anatomy, University of Regensburg, Universitätsstr. 3193053 Regensburg (Germany) E-mail: ralph.witzgall@vkl.uni-regensburg.de

**Keywords:** cyanines, electron, fluorescence, microscopy, readout

Imaging is a key research goal for the understanding of biological processes by proteins or nucleic acids inside the living cell.[1–3] By using classical confocal fluorescence microscopy it is possible to track those biomolecules that are labeled covalently with a suitable fluorophore[4, 5] or fluorescent protein.[6] Fluorescence microscopy as a noninvasive and nondestructive method (if the applied fluorophores are sufficiently photostable) offers the advantage to follow biological processes in real time over hours or even days. Over the last two decades, advanced fluorescence spectroscopy methods, such as stimulated emission depletion (STED),[7] photoactivated localization microscopy (PALM)[8] and others, have revitalized light-based microscopy for cell biology. Additionally, cell images obtained by electron microscopy allow the characterization of subcellular structures. The most complete picture would be drawn from imaging, if dynamic fluorescence and static electron microscopy were combined and correlated by applying a single label that gives readout in both types of microscopy. So far, this has been achieved to a certain extent for observing and localizing lipids and proteins inside cells but not for nucleic acids.

The idea to use fluorescent chromophores not only for fluorescence microscopy but additionally as chromogenes to stain electron microscopy images has been published for the first time in 1982 to image neurons.[9] Since then, a variety of examples were presented mainly for proteins.[10–14] The most successful technique is to use the fluorophores or fluorescent proteins themselves in order to photooxidize 3,3′-diaminobenzidine (DAB) which initiates polymerization.[9, 10, 12, 15–17] The resulting polymer can be stained by heavy atom salts, such as OsO_4_, and finally be visualized in histological sections by electron microscopy. Proteins were covalently and noncovalently labeled with biarsenical fluorophores,[10] fluorescent proteins,[12, 17] 4′,6-diamidino-2-phenylindole (DAPI), eosin,[13] lucifer yellow[16] propidium iodide[16, 18] or quantum dots[11] to get specific readout not only in confocal fluorescence spectroscopy but also in electron microscopy. To our knowledge, however, the methodology by photoinduced DAB polymerization has never been transferred to image nucleic acids. There is only one example of staining nucleic acids in electron micrographs, which, however, uses a completely different technique.[19] The goal of this work was to identify labels that enable a readout in both types of microscopy. Herein, we present the photochemical evaluation of three different fluorophores covalently attached to short DNA and RNA strands and their ability to image nucleic acids inside cells both by confocal microscopy and by well-resolved snapshots taken by electron microscopy. We chose representatively the delivery process of nucleic acids into cells to test the dual readout of our synthetic DNA– and RNA–conjugates in both imaging methods. It is important to mention here that we recently have shown that similarly modified siRNA exhibits only slightly reduced functionality in comparison to non-modified RNA.[20]

Although the mechanism of photochemically induced polymerization of DAB is not completely understood, recent studies with eosin[21] and other chromophores[16, 17] revealed that singlet oxygen (^1^O_2_) and oxygen radicals (such as the superoxide anion O_2_^.−^) may play a key role as initiators of photopolymerization. These reactive oxygen species are formed in situ upon excitation of chromophores. Direct photooxidation has been identified as an alternative pathway,[21] since DAB is a good electron donor (*E*_ox_=0.335 V vs. Ag^+^/AgCl).[21, 22] Using a conversion constant of ca. +0.55 V[23] from the Ag^+^/AgCl electrode to the normal hydrogen electrode (NHE), DAB exhibits an oxidation potential of approximately 0.9 V vs. NHE. Hence, we identified for our studies three fluorescent chromophores with suitable redox potentials and excitation energies that are sufficient for direct photooxidation of DAB. These are (1) perylene (Pe) conjugated to the 5-position of 2′-desoxyuridine in DNA1, (2) cyanine indole quinolinium (CyIQ) attached to the 2′-position of uridine in DNA2 and DNA3,[24] and (3) thiazole orange (TO) as a base substitution in DNA4 and RNA5 (Figure 1; Table 1).[25–27] The latter chromophore (TO), a fluorophore well known from noncovalent staining agents like TOTO,[28] shows a reduction potential of −1.0 V (vs. NHE).[29] According to the Rehm–Weller equation Δ*G*=*E*_ox_−*E*_red_−*E*_00_ (without the Coulomb contribution) and *E*_00_=2.4 eV for TO, the driving force Δ*G* for DAB photooxidation by DNA4 and RNA5 is approximately −0.5 eV. The driving force for DNA2 and DNA3 is a little higher (Δ*G*=−0.7 V) since CyIQ exhibits a slightly different *E*_red_=−0.8 V (vs. NHE; see Supporting Information) but identical *E*_00_=2.4 eV. Pe in DNA1 represents the weakest electron acceptor (Δ*G*=−0.3 V) in this set of chromophores with *E*_red_=−1.5 V (see Supporting Information) and *E*_00_=2.7 eV.

**Figure 1:**
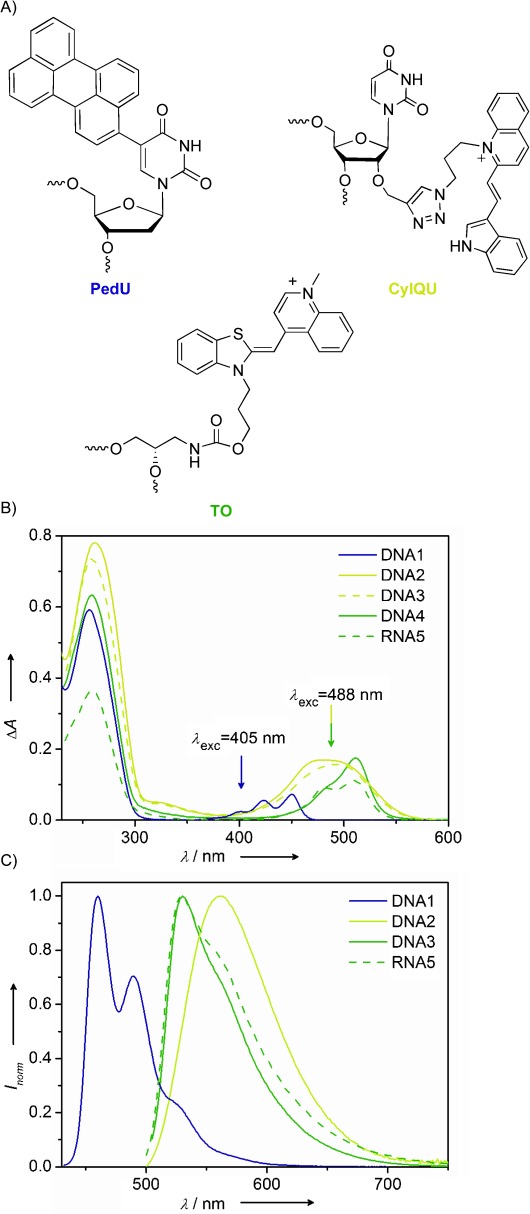
A) Structure of fluorophore–DNA conjugates: perylene attached to the 5-position of 2′-desoxyuridine (PedU), cyanine indole quinolinium attached to the 2′-position of uridine (CyIQU) and thiazole orange as a DNA/RNA base substitution (TO). B) UV/Vis absorption. C) Fluorescence of DNA1–DNA4 and RNA5 at 2.5 μm in NaH_2_PO_4_/NaH_2_PO_4_ (50 mm, pH 7), NaCl (250 mm), 25 °C, *λ*_exc_=426 nm (Pe), 490 nm (TO) or 495 nm (CyIQ).

**Table 1:** Photochemical data of the chromophore labels and sequences of oligonucleotides DNA1–DNA4, RNA5

DNA	Label	*E*_red_ [V]	*E*_00_ [eV]	Δ*G*^[a]^
	Pe	−1.5	−2.7	−0.3 eV
DNA1: 5′-(**PedU**)TA CTG TGA CTG ATG CTA TGA CGC A-3′				
	CyIQ	−0.8	−2.4	−0.7 V
DNA2: 5′-AGC TTA CCG CG(**CyIQU**) ATT TCA A(**CyIQU**)C GTA CCG G-3				
DNA3: 5′-GAT CCC GGT ACG A(**CyIQU**)T GAA A(**CyIQU)**A CGC GGT A-3′				
	TO	−1.0	−2.4	−0.5 eV
DNA4: 5′-(**TO**)TA CTG TGA CTG ATG CTA TGA CGC A-3′				
RNA5: 5′-gca guc uu(**TO**) uuc acug a-3′				

[a] For photoinduced DAB oxidation according to Δ*G*=*E*_ox_−*E*_red_−*E*_00_.

The fluorophore–DNA/RNA conjugates PedU, CyIQU and TO were synthesized according to our published protocols and incorporated into oligonucleotides either using the corresponding phosphoramidites[27] or by postsynthetic “click”-type cycloaddition.[24] All synthetic oligonucleotides were purified by semi-preparative HPLC and identified by mass spectrometry (MALDI-TOF or ESI). To complete the photochemical characterization, optical spectroscopy was applied. UV/Vis absorption of the three single-stranded oligonucleotides reveals the presence of the chromophores between 400 nm and 550 nm with maxima at 450 nm (Pe in DNA1), 480 nm (CyIQ in DNA2/DNA3) and 510 nm (TO in DNA4/RNA5). Fluorescence occurs with Stokes’ shifts of 10 nm (460 nm, Pe in DNA1), 20 nm (530 nm, TO in DNA4/RNA5) and 80 nm (560 nm, CyIQ in DNA3/DNA4). Especially the latter two values should be sufficiently high to separate excitation from emission wavelengths in confocal microscopy.

As already pointed out above, the delivery process of nucleic acids into cells was representatively chosen to test the readout of the synthetic DNA– and RNA–conjugates. In order to demonstrate that the DNA conjugates can be visualized by both light and electron microscopy, LLC-PK1 cells, a porcine kidney epithelial cell line, were transfected with the respective modified nucleic acids. When LLC-PK1 cells were transiently transfected with a complex consisting of the respective nucleic acid and Lipofectamine 2000, positive cells could be easily visualized already 1 hour after the addition of the nucleic acids formulation. Fluorescence microscope images show round structures of various sizes which could be identified in the cytoplasm (Figure 2). At this stage, no obvious differences could be detected between single- and double-stranded oligonucleotides (DNA2 versus DNA2/DNA3) (see the Supporting Information).

**Figure 2:**
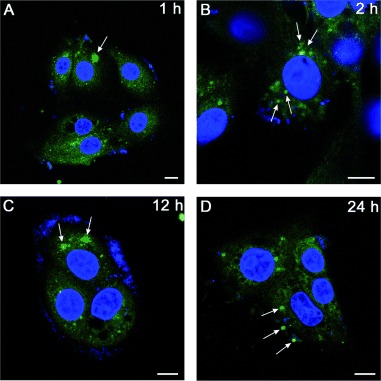
Fluorescently labeled oligonucleotides are taken up by transiently transfected LLC-PK1 cells. LLC-PK1 cells were transfected with single-stranded oligonucleotide DNA2 (stock concentration: 100 μm) and Lipofectamine 2000. Fluorescent cytosolic structures (arrows) are shown A) 1 h, B) 2 h, C) 12 h and D) 24 h after the addition of the DNA/lipofectamine mixture (white bar represents 10 μm).

The strong fluorescence in chemically fixed cells encouraged us to trace the delivery of fluorescently labeled oligonucleotides by live-cell microscopy. Indeed, especially the fluorescent label CyIQ in DNA2 (and DNA3) was stable enough to be followed over an extended period of time. Hence, we concentrated our subsequent work on DNA2. We were able to observe how aggregates of nucleic acids moved in the medium and were then taken up by the cells. In order to better understand by what mechanism the nucleic acids were taken up, the fixed cells were immunostained with antibodies against early endosome antigen 1 (EEA1), a marker for early endosomes, and lysosomal-associated membrane protein 1 (LAMP1), a marker for lysosomes. We were able to detect the fluorescently labeled oligonucleotides both in EEA- and in LAMP1-positive structures, indicating that the oligonucleotides were taken up by endocytosis and were delivered to lysosomes (Figure 3). No strong fluorescence was detected in the nuclei of transfected cells.

**Figure 3:**
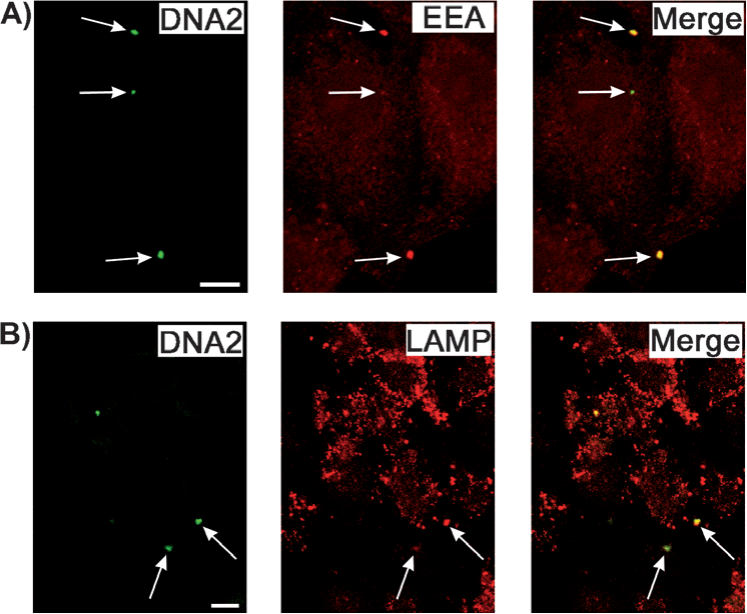
Fluorescent oligonucleotides are present in early endosomes and in lysosomes. LLC-PK1 cells were transfected with single-stranded oligonucleotide DNA2 (stock concentration: 100 μm) and Lipofectamine 2000. Two hours after the addition of the DNA/lipofectamine mixture, the cells were stained with primary antibodies against A) EEA1 and B) LAMP1, markers for early endosomes and lysosomes, respectively. The arrows point to EEA1- and LAMP1-positive structures containing fluorescent oligonucleotides (white bar represents 10 μm).

The main goal of this study was to apply the synthetic fluorophore–DNA/RNA conjugates in order to obtain a readout in cellular snapshots analyzed by transmission electron microscopy. Based on their photochemical properties as characterized above, all three fluorescent dyes (Pe, CyIQ and TO) have the potential to localize the labeled nucleic acids on an ultrastructural level by photoinduced DAB polymerization. As already explained above, exposure to an intense light beam leads to the generation of DAB radical cations and/or radical oxygen species, which in turn generate polymerized DAB. By staining with heavy metal salts, polymerized DAB can be readily visualized with high resolution in the electron microscope. Hence, photooxidized cells were identified by a brown precipitate that allowed their subsequent imaging and localization of the delivered nucleic acid by transmission electron microscopy. In these experiments, we obtained the best results with the cyanine-based fluorescent dye CyIQ (Figure S1 in the Supporting Information). This correlates well with our recent finding that CyIQ represents a remarkably photostable fluorescent dye.[24] With this technique, we were able to visualize the delivered DNA and RNA in distinct subcellular structures with astonishing resolution by taking snapshots of transfected cells. In some cells, the plasma membrane was decorated by a brown precipitate (Figure 4 A,B) indicating the penetrating nucleic acids. The cytosol contained round structures in which no further details could be seen due to the heavy precipitate. In the case of other cytosolic structures, brown precipitates and thus delivered nucleic acids were detected in membrane-lined vesicles together with other small vesicles (Figure 4 C). These could therefore be identified as multivesicular bodies (i.e., late endosomes), which corroborated our immunofluorescence staining for EEA1 and LAMP1. Only in very rare cases did we find electron-dense precipitates in the nuclei (Figure 4 D,E), indicating the absence of nucleic acid delivery therein. This result is supported by fluorescence imaging as described above and shows nicely that both imaging techniques can be combined. However, it is noteworthy that fixation of the cells can change the internal distribution of the internalized oligonucleotides to some extent. Hence the correlation of readout by fluorescence microscopy and by electron microscopy is difficult at this stage. It is important to mention that control experiments without the covalently attached redox-active labels did not yield readout and thus did not image any nucleic acids in the plasma membrane, nucleus and multivesicular bodies (Figure S2 in the Supporting Information).

**Figure 4:**
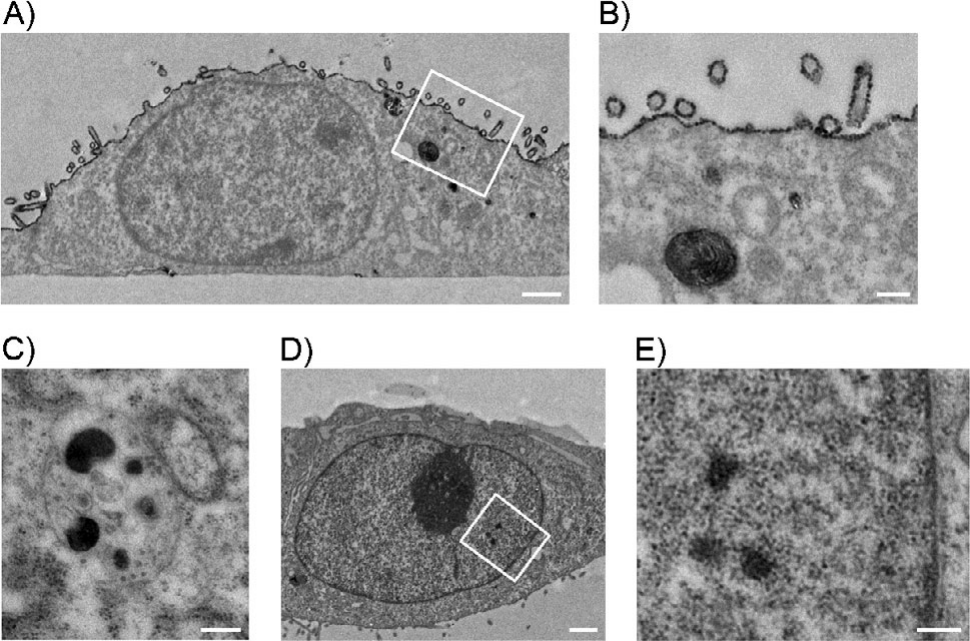
Electron microscopic detection of internalized oligonucleotides. LLC-PK1 cells were transfected with single-stranded DNA oligonucleotide DNA2 (stock concentration: 100 μm) and Lipofectamine 2000. Fluorescent cells were photooxidized in the presence of DAB. In some cells, a labeling of the plasma membrane could be detected (A,B). A membrane-lined cytosolic structure contains electron-dense precipitates and additional small vesicles, and therefore can be identified as a late endosome (C). Few nuclei contained electron-dense structures that could be clearly differentiated from heterochromatin (D, E). The chromatin staining in D) seems to be an artifact. White bar represents 1000 nm (A,B,D) or 250 nm (C,E).

The photochemically active chromophore–nucleic acid conjugates presented herein allow not only imaging by fluorescence microscopy but additionally are able to photoinduce DAB polymerization as staining for electron microscopy. The well-known lipofectamine-dependent delivery process of nucleic acids into cells was chosen to test synthetic DNA and RNA conjugates in both imaging methodologies and to identify labels that are able to give readout in both types of microscopy. The photochemical characterizations show clearly that TO, CyIQ and Pe as fluorophore–DNA conjugates and TO as a fluorophore–RNA conjugate can be applied. Their optical properties provide readout in fluorescence microscopy, and their redoxactive properties enable readout in electron microscopy. Especially the CyIQ dye in DNA2 (and DNA3) behaves very photostable[24] and allows the observation of cellular uptake of DNA and RNA into LLC-PK1 cells by confocal fluorescence microscopy over hours or even days. Moreover, electron microscopic images can be obtained by using the redox activity of the same labels. Photochemically induced polymerization occurs only where the nucleic acid is located, since the fluorophores have been attached covalently to DNA. This photochemical process allows exclusive staining of nucleic acids; the electron microscope images show good contrast and astonishing resolution in order to distinguish subcellular structures in transfected cells. Now, it is possible to use transmission electron microscopy to localize delivered nucleic acids on an ultrastructural level and thus to gain more detailed insights into the delivery processes. These experiments showed that the cyanine-based fluorescent dye again gave the best contrasts. The focus of this study was to provide a proof-of-concept for the photoselective staining of nucleic acids in electron microscopy. Further studies to prove the functionality of the labeled DNA and RNA constructs will be performed in the future. At the moment, we refer here to our recent work on TO-labeled siRNA that showed only slightly reduced silencing activity due to the TO label.[20] In conclusion, it became clear that the most complete picture will arise, if dynamic fluorescence microscopy can be combined with static electron microscopy by labels that give readout in both types of microscopy. The presented concept of combining the optical with the photoredox properties of chromophores is astonishingly simple and should be applicable, in principle, to every RNA and DNA sequence in any biological cell of interest.

## Experimental Section

Full details of the experimental procedures can be found in the Supporting Information.

**Oligonucleotide preparation**: The fluorophore conjugates with PedU, CyIQU and TO were synthesized chemically using the corresponding phosphoramidites or by postsynthetic “click”-type cycloaddition, purified by HPLC and identified using MS (Table S1, Figure S3–S7 in the Supporting Information).

**Transfection of LLC-PK1 cells and electron microscopy**: The day before transfection ∼2×10^5^ LLC-PK1 cells were plated into 35 mm μ-dishes. For transfection, fluorescently labeled oligonucleotides of the indicated concentrations were combined with Lipofectamine 2000 and serum-free medium. After the cells were fixed overnight, 3,3′-diaminobenzidine (DAB) was added, and fluorescent cells were exposed to light of 585 nm wavelength. The cells were washed again and contrasted with OsO_4_ and uranyl acetate. Sections were prepared with an ultramicrotome.

**Fluorescence imaging.** For live-cell imaging, LLC-PK1 cells were transfected in 35 mm μ-dishes; for (immuno)fluorescence imaging cells, were transfected on glass cover slips. Live-cell imaging was performed using an excitation wavelength of 488 nm and by collecting photons between 493 and 598 nm. Pictures were taken every 3 min for a total period of ∼6 h.

## Supplementary material

As a service to our authors and readers, this journal provides supporting information supplied by the authors. Such materials are peer reviewed and may be re-organized for online delivery, but are not copy-edited or typeset. Technical support issues arising from supporting information (other than missing files) should be addressed to the authors.
