# Supplementary material for: Photochemically Active Fluorophore–DNA/RNA Conjugates for Cellular Imaging of Nucleic Acids by Readout in Electron Microscopy
Source: ChemistryOpen. 2013 Jun 21;2(4):136–40. doi: 10.1002/open.201300017 (PMC3775519; doi:10.1002/open.201300017)
Supplement: Supplementary file 1 [file open0002-0136-SD1.pdf]

ChemistryOPEN

Supporting Information

© 2013 The Authors. Published by Wiley-VCH Verlag GmbH & Co. KGaA, Weinheim

Photochemically Active Fluorophore–DNA/RNA Conjugates for Cellular Imaging of Nucleic Acids by Readout in Electron Microscopy

Carolin Holzhauser,[a] Sabrina Kracher,[b] Moritz M. Rubner,[a] Wolfgang Schmucker,[a] Hans-Achim Wagenknecht,*[a] and Ralph Witzgall*[b]

open_201300017_sm_miscellaneous_information.pdf

Spectroscopic measurements were recorded in Na-P*i* buffer solution (10 mM, pH = 7) with 250 mM NaCl using quartz glass cuvettes (10 mm). Absorption spectra were recorded with a Varian Cary 100 spectrometer equipped with a 6x6 cell changer unit at 20 °C. Fluorescence was measured with a Jobin–Yvon Fluoromax 3 fluorimeter with a step width of 1 nm and an integration time of 0.2 s. All spectra were recorded at 20 °C and with an excitation and emission band pass of 3 nm and are corrected for Raman emission from the buffer solution. ESI mass spectra were measured in the central analytical facility of the Institute of Organic Chemistry of the University of Regensburg on a ThermoQuest Finnigan TSQ 7000 in negative and positive ionization mode. BIFLEX-IVMALDI-TOF mass spectra were measured on a Bruker Daltonics with 2,4,6-trihydroxyacetophenone (0.3 M in EtOH) and diammoniumcitrate (0.1 M in H_2O), ratio 2:1, as matrix. NMR spectra were recorded on a Bruker Avance 300 spectrometer in deuterated solvents (^{1}H at 300 MHz, ^{13}C at 75 MHz). Chemical shifts are given in ppm relative to TMS.

Transfection of LLC-PK1 cells and electron microscopy

The day before the transfection ~2x 105 LLC-PK1 cells were plated into 35-mm µ-Dishes (ibidi GmbH, Martinsried, Germany). For transfection, 1 µl of fluorescently labelled oligonucleotides of the indicated concentrations were combined with 2 µl of Lipofectamine 2000 and 497 µl of serum-free medium. For longer incubation periods, the transfection mixture was removed after 4 hours and replaced by DMEM/10% FCS. After the indicated period of time, cells were fixed with 1x PBS, 2% glutaraldehyde for 5 minutes at room temperature and then overnight at 4°C. Following 5 2-minute washes with 100 mM Na cacodylate pH 7.4, 1 ml of ice-cold 100 mM Na cacodylate pH 7.4, 1 mg/ml of diaminobenzidine was added and fluorescent cells were exposed for 15-20 minutes 4°C to light of 585 nm wavelength. The cells were washed again with 100 mM Na cacodylate pH 7.4, contrasted with 1% OsO4 and 2% uranyl acetate, and then embedded in Durcupan. 50 nm sections were prepared with an ultramicrotome and pictures taken with a Zeiss EM 902 transmission electron microscope equipped with a cooled CCD digital camera (TRS, Moorenweis, Germany).

Fluorescence imaging

For live-cell imaging LLC-PK1 cells were transfected in 35-mm µ-Dishes, for (immuno)fluorescence imaging cells were transfected on glass cover slips. Live-cell imaging was performed on a Zeiss LSM 710 confocal laser scanning microscope with an excitation wavelength of 488 nm and by collecting photons between 493 and 598 nm. Pictures were taken every 3 minutes for a total period of ~6 hours. For (immuno)fluorescence imaging cells were fixed for 20 minutes at room temperature with 1x PBS/4% paraformaldehyde. If no immunostaining was performed, the cells were washed 3 times 5 minutes with 1x PBS, stained for 1 minute with 10 µg/ml of Hoechst 33258 and mounted with 1x PBS/40% glycerol. For immunostaining, cells were washed 3 times 5 minutes with 1x PBS, and blocked with 1x PBS, 2% BSA, 0.1% Triton-X 100 for 1 hour at room temperature. Then the cells were washed 5 minutes with 1x PBS and incubated with the primary antibody overnight at 4°C. The cells were washed 4 times 5 minutes with 1x PBS/350 mM NaCl and once with 1x PBS before the secondary antibody was added for 1 hour at room temperature. The following primary antibodies were used: The mouse monoclonal anti-EEA1 antibody 14/EEA1 (BD Transduction Laboratories, cat. nr. 610456; diluted 1:200), a rabbit polyclonal anti-LAMP-1 antibody (Sigma, cat. nr. SAB3500285; diluted 1:50). The following secondary antibodies were used: A DyLight 405-conjugated donkey polyclonal anti-rabbit IgG (Rockland, cat. nr. 611 746 127; diluted 1:1,000), and a DyLight 405-conjugated donkey polyclonal anti-mouse IgG (Rockland, cat. nr. 610 746 124; diluted 1:1,000). Pictures were taken with a Zeiss LSM 710 confocal laser-scanning microscope.

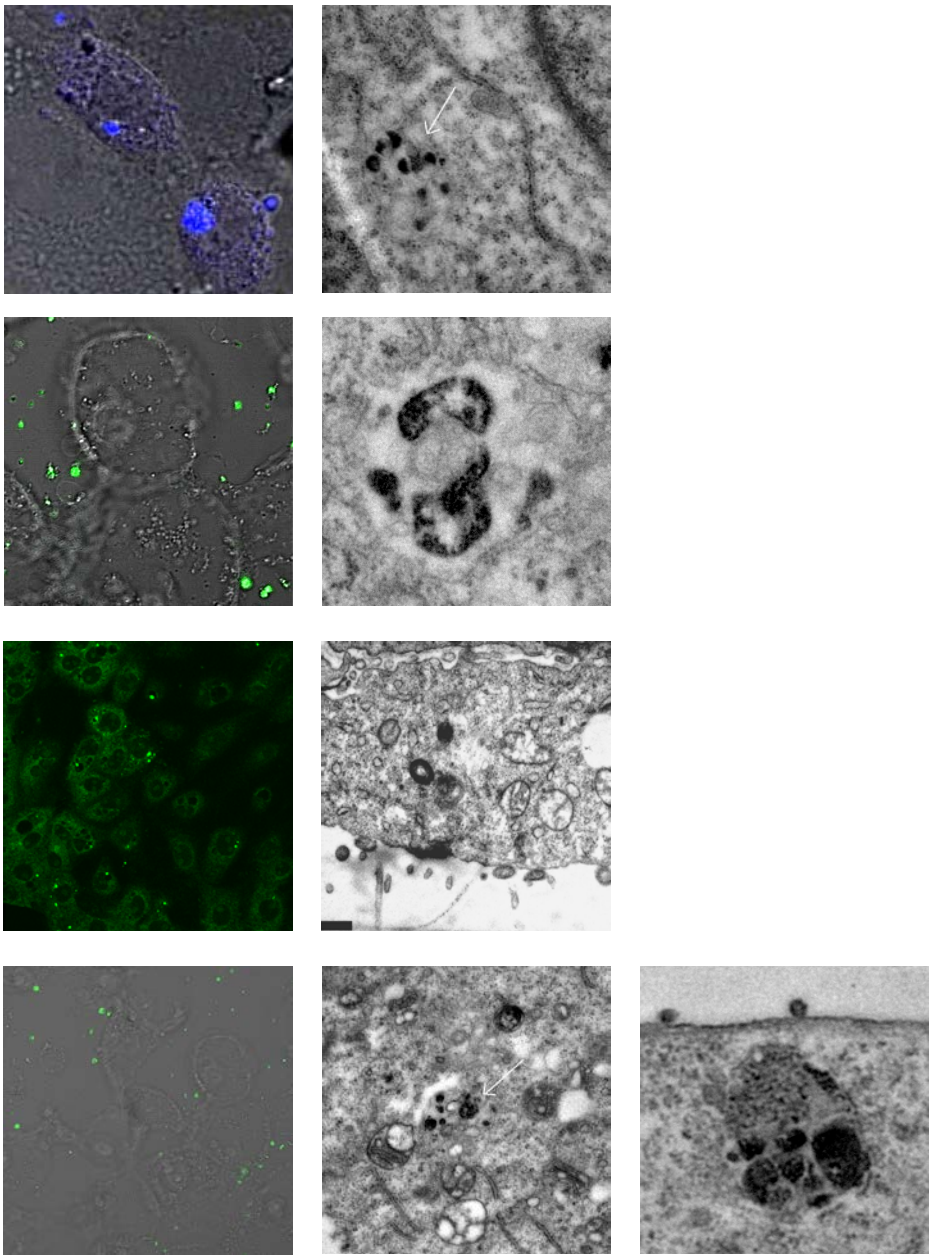

Figure S1: Combined light and electron microscopy. LLC-PK1 cells were transfected with the single-stranded oligonucleotide (stock concentration: 100 µM) and lipofectamine 2000. Left: Confocal light microscopy reveals endosomes taken up by the cells (excitation at 405 nm for DNA1, 488 nm for the others); right: Fluorescent cells were photooxidized in the presence of diaminobenzidine. Endosomes are identified by increased contrast.

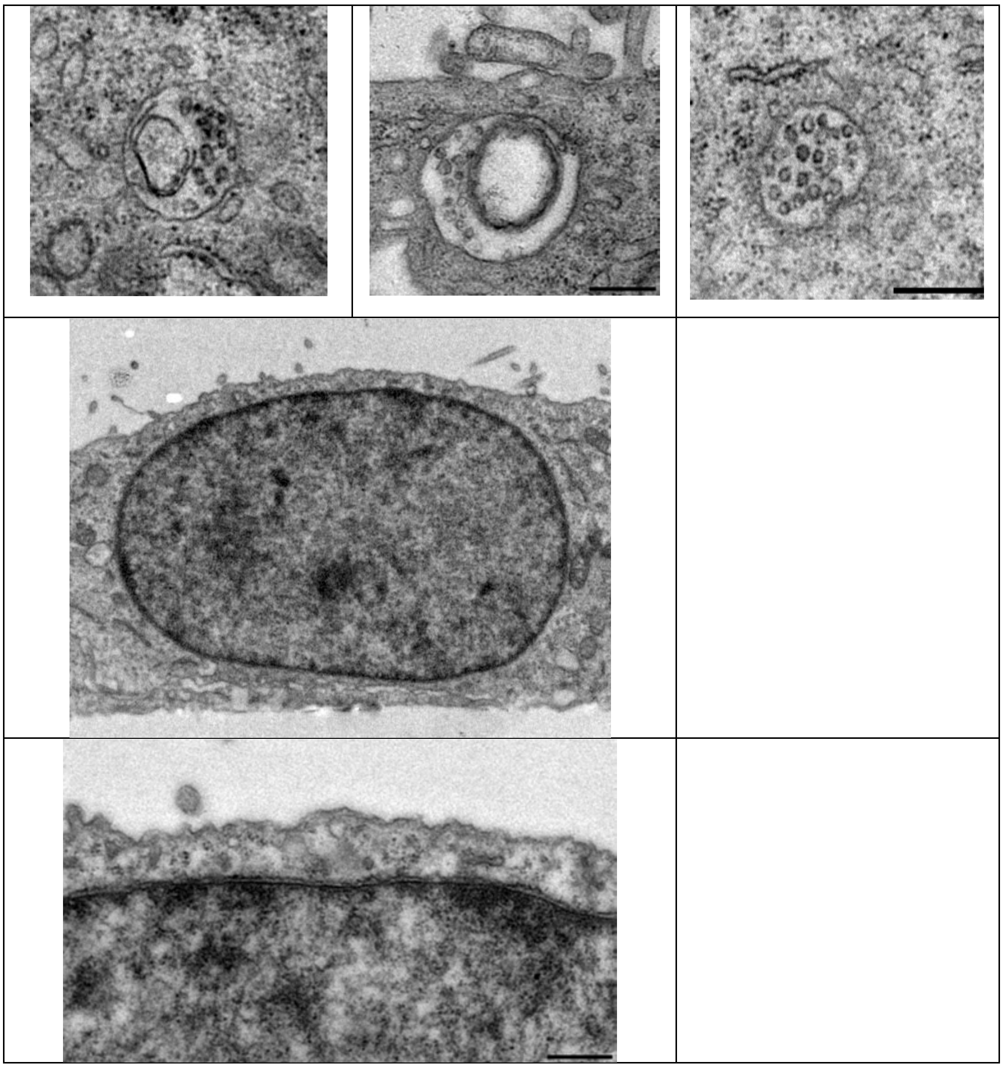

Figure S2: Control images without redoxactive labels of nucleus and extra-vesicular bodies (top) and plasmamembrane (bottom).

Preparation of modified oligonucleotides

Oligonucleotides were prepared on an Expedite 8909 Synthesizer from Applied Biosystems (ABI) using standard phosphoramidite chemistry. Reagents and controlled pore glass (CPG) (1 µmol) were purchased from ABI and Glen Research. The 2´propargyl-uridine (cU) was purchased from ChemGenes. The fluorophore-conjugates PedU, CyIQU and TO were synthesized according to our published protocols and incorporated into oligonucleotides either using the corresponding phosphoramidites or by postsynthetic click-type cycloaddition.[1,2] After preparation, the oligonucleotides were cleaved from the resin and deprotected by treatment with conc. NH_4OH at room temperature for 24 h. The modified oligonucleotides were purified by HPLC on a semi preparative RP-C18 column (300 Å, Supelco) using the following conditions: A) NH_4OAc buffer (50 mM), pH) 6.5; B) acetonitrile; gradient 0 – 20 % B over 45 min, flow rate 2.5 mL/min, UV/vis detection at 260 nm, 290 nm and 495 nm. Purification was verified by LC-MS (ESI) or MALDI-TOF-MS. Finally the oligonucleotides were lyophilized and quantified by their absorbance in 10 mM sodium phosphate buffer at 260 nm on a Varian Cary 100 spectrometer.

Table S1: ESI MS[(a)] and MALDI MS[(b)] data and extinction coefficients of oligonucleotides in **DNA1-DNA4** and **RNA5**.

**Sample**	**calculated [g/mol]**	**found [g/mol]**	**ε_{260} [L*mol^{-1}*cm^{-1}]**
**DNA1 (PedU)**[(a)]	7904.8	7905.6	247200
**DNA2 (CyIQU)**[(b)]	9324.7	1555.05 $[M^{2+}+4H]^{6+}$	265000
		1865.7 $[M^{2+}+3H]^{5+}$	
**DNA3 (CyIQU)**[(b)]	9422.7	1571.6 $[M^{2+}+4H]^{6+}$	270000
		1885.2 $[M^{2+}+3H]^{5+}$	
**DNA4 (TO)**[(a)]	7892.8	7889.7	234600
**RNA5 (TO)**[(a)]	5545.2	5544.3	147000

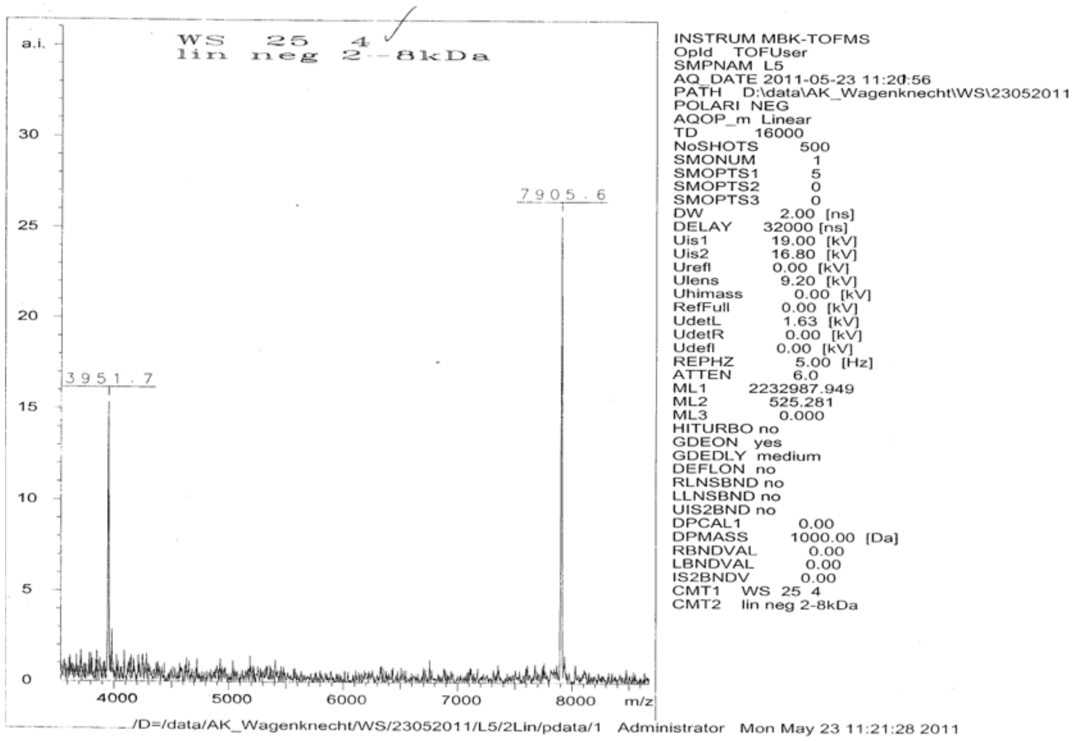

Figure S3. Image of ESI MS analysis of **DNA1 (PedU)**.

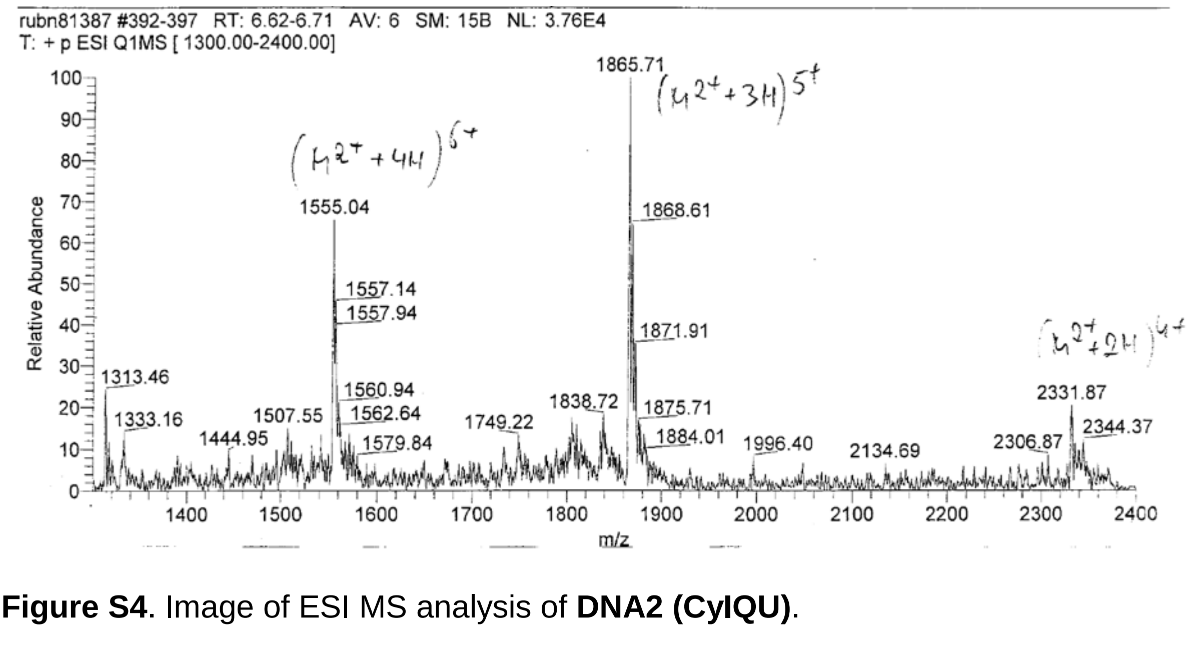

Figure S4. Image of ESI MS analysis of **DNA2 (CyIQU)**.

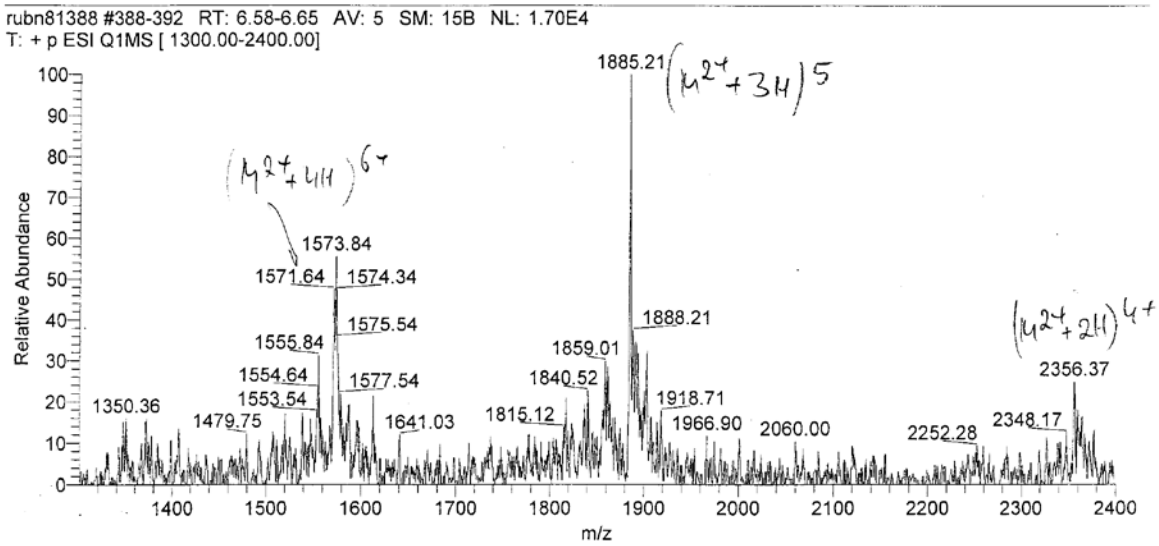

Figure S5. Image of MALDI MS analysis of **DNA3 (CyIQU)**.

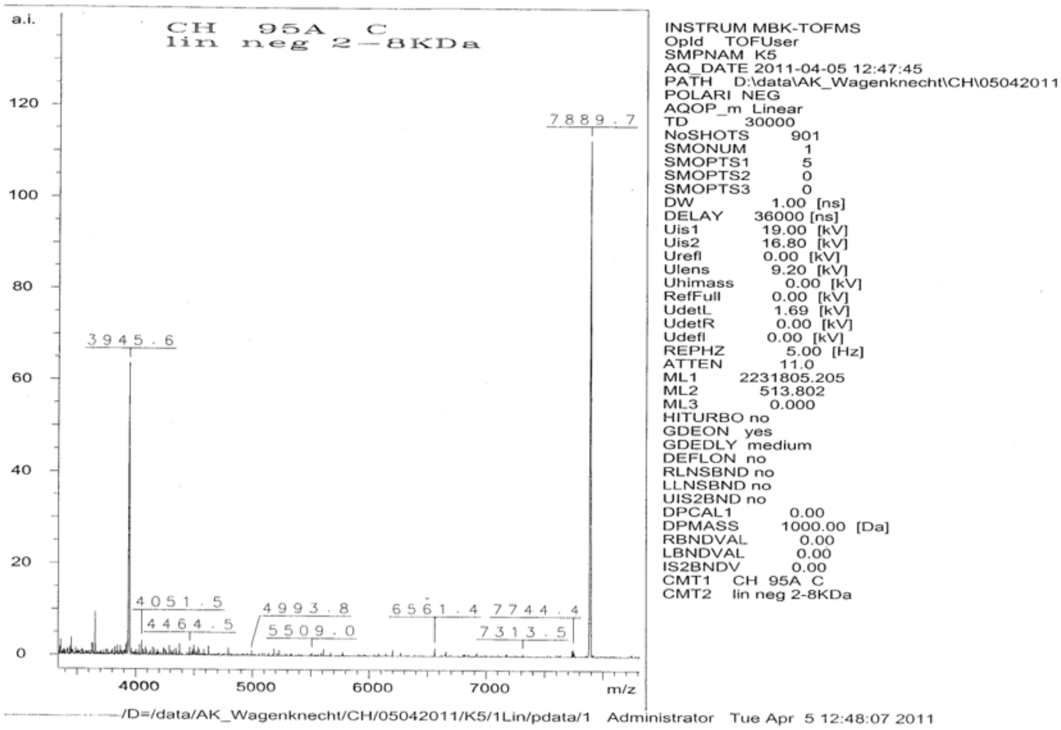

Figure S6. Image of ESI MS analysis of **DNA4 (TO)**.

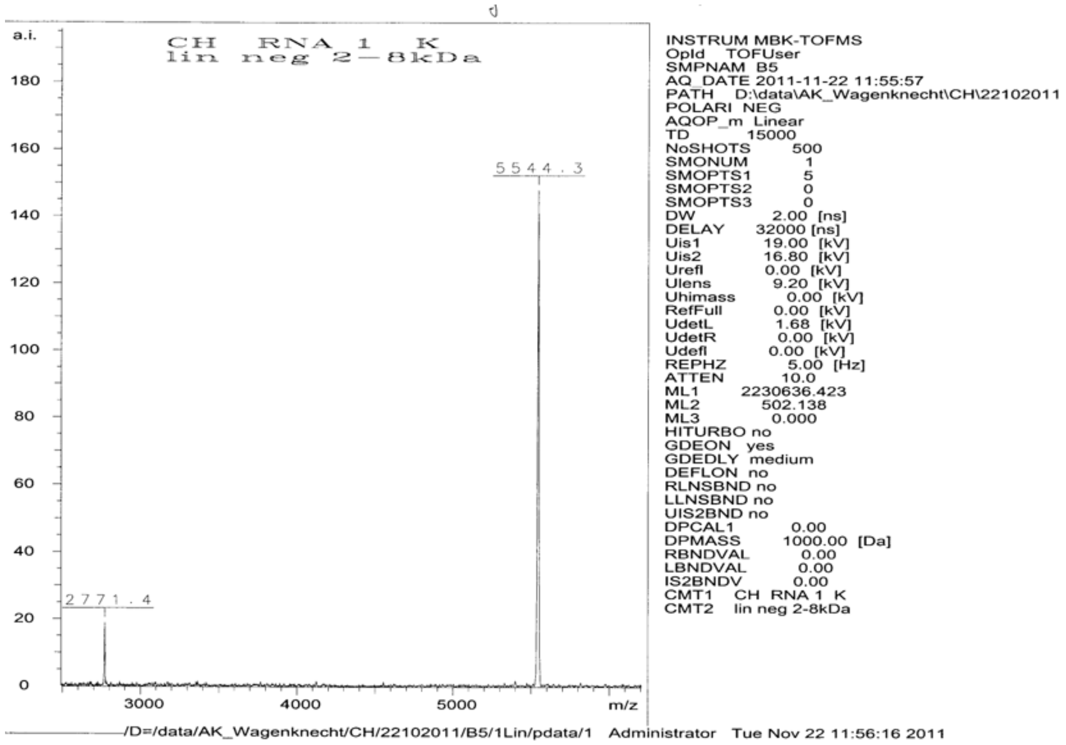

Figure S7. Image of MALDI-TOF MS analysis of **RNA5 (TO)**.

Synthesis of the PedU DNA building block

Synthesis of 5-(perylen-3-yl)-2´-desoxyuridin (1)

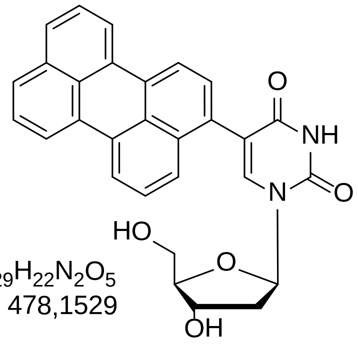

$C_{29}H_{22}N_2O_5$
M: 478,1529

280 mg (0.74 mmol) 4,5,5-tetramethyl-2-(perylen-3-yl)-1,3,2-dioxaborolan[3,4,5] and 247 mg (0.70 mmol) 5-iodo-2´-desoxyuridine were dissolved in 80 mL of THF/H_2O/MeOH 2:1:1. 53.0 mg (65.7 mmol) Pd(dppf)Cl_2 and 399 mg (9.98 mmol, 13.5 eq.) NaOH were added. The mixture was stirred at 65 °C for 18 h, the pH was adjusted to 7 by 2 N HCl, and the reaction mixture was extracted several times by EtOAc. The combined organic layers were dried with Na_2SO_4 and dried under vacuum. The crude product was purified by column chromatography (EtOAc:MeOH 50:1) yielding 124 mg (0.26 mmol, 37%) as yellow foam.

^{1}H NMR (300 MHz, d_6-DMSO): δ [ppm] 11.59 (s, 1H); 8.38 (m, 4H); 8.10 (s, 1H); 7.82 (d, J = 8.2 Hz, 2H); 7.56 (m, 4H); 7.41(d, J = 7.7 Hz, 1H); 6.28 (t, J = 6.6 Hz, 1H); 5.26 (d, J = 4.1 Hz, 1H); 4.89 (t, J = 4.9 Hz 1H); 4.25 (m, 1H); 3.79 – 3.50 (m, 2H); 2.21 (m, 2H)

^{13}C NMR (300 MHz, d_6-DMSO): δ [ppm] 162.4; 150.5; 149.5; 139.6; 136.8; 134.3; 133.4; 131.4; 130.7; 130.6; 130.6; 130.3; 129.2; 128.1; 128.1; 128.0; 127.8; 127.0; 126.8; 126.3; 120.9; 120.9; 120.3; 113.7; 87.5; 84.6; 70.4; 61.1.

ESI MS m/z (%): 478.2 [M^+].

HR-ESI-MS m/z (calc.) = 478.1529 [M^+], m/z (found) = 478.1536 [M^+].

Synthesis of 5'-*O*-((bis(4-methoxy-phenyl)-phenyl-methoxy)-5-(perylen-3-yl)-2´-desoxyuridin (2)

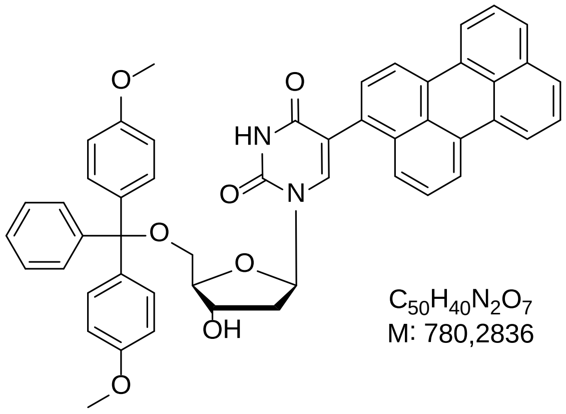

144 mg (0.30 mmol) **1** were dissolved in 15 mL dry pyridine and 800 mL NEt_3. 1.03 g (3.05 mmol) DMT-Cl were added. The mixture was stirred at r.t. for 18 h. The solvent was removed under vacuum and the crude product was purified by column chromatography (EtOAc:THF 10:1) yielding 193 mg (0.25 mmol, 82%) as yellow foam.

^{1}H NMR (600 MHz, d_6-DMSO): δ [ppm] 11.62 (s, 1H); 8.39 (d, J = 7.9 Hz, 2H); 8.20 (br m, 1H); 7.80 (d, J = 8.1 Hz, 2H); 7.69 (br m, 1H); 7.62 – 7.38 (m, 3H); 7.15 – 7.11 (m, 5H); 6.96 – 6.78 (m, 5H); 6.58 – 6.48 (m, 4H); 6.30 (br m, 1H); 5.39 (br, m, 1H); 4.53 (s, 1H); 3.94 (br m, 1H); 3.56 (s, 3H); 3.53 (s, 3H); 3.25 (br m, 1H); 2.44 (br m, 1H); 2.31 (s, 1H).

^{13}C NMR (600 MHz, d_6-DMSO) δ [ppm] 162.3; 157.9; 157.9; 157.9; 150.3; 138.5; 134.3; 133.5; 130.6; 130.6; 130.3; 129.6, 129.6; 129.5; 129.0; 128.1; 128.0; 128.0; 127.9; 127.8; 127.3; 127.2; 127.0; 127.0; 126.5; 126.3; 120.9; 120.8; 120.6; 120.0; 113.0; 112.9; 86.0; 54.9; 54.9.

ESI MS m/z (%): 779.1 (100) [$M-H^+$].

HR ESI-MS m/z (calc.) = 780.2830 [M^+], m/z (found): 780.2837 [M^+].

Synthesis of 3’-O-((2-Cyanoethoxy)(diisopropylamino)phosphinyl)-5’-O-((bis(4-Methoxy-phenyl)-phenyl-methoxy)-5-(perylen-3-yl)-2´-desoxyuridin (3)

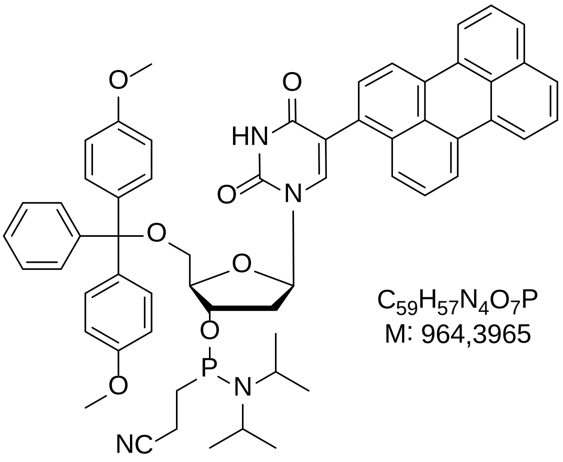

192 mg (0.25 mmol) **2** were dissolved in 10 mL dry CH_2Cl_2 130 µL (0.76 mmol) $EtN(iPr)_2$ and 110 µL (117 mg, 0.5 mmol) 2-cyanoethyl-*N,N*-diisopropylchloro-phosphoramidit were added. After 2 h the product was purified by column chromatography (THF:EtOAc:pyridine 10:100:1) yielding 238 mg (0.25 mmol, 98%) as yellow foam.

^{31}P NMR (300 MHz, $CDCl_3$): δ [ppm] 148.5; 148.3.

References

1. C. Holzhauser. S. Berndl, F. Menacher, M. Breunig, A. Göpferich, H.-A. Wagenknecht, *Eur. J. Org. Chem.* **2010***,* 1239-1248.

2 M. M. Rubner, C. Holzhauser, P. B. Bohländer, H.-A. Wagenknecht, *Chem. Eur. J.* **2012**, 18, 1299-1302.

3 C. Beyer, H. A. Wagenknecht, *J. Org. Chem.* **2010**, *75*, 2752-2755.

4 H. Maeda, Y. Nanai, K. Mizuno, J. Chiba, S. Takeshima, M. Inouye, *J. Org. Chem.* **2007**, 72, 8990-8993.

5 Y. Avlasevich, C. Kohl, K. Mullen, *J. Mater. Chem.* **2006**, *16*, 1053-1057.
